# Editorial: Microbial hydrocarbon degradation and bioremediation: from genes to pathways

**DOI:** 10.3389/fmicb.2024.1416516

**Published:** 2024-04-26

**Authors:** Lucymara Fassarella Agnez-Lima, Marilene Henning Vainstein, Xuwang Zhang

**Affiliations:** ^1^Departament of Cellular Biology and Genetics, Center of Biosciences, Federal University of Rio Grande do Norte, Natal, RN, Brazil; ^2^Centro de Biotecnologia, Universidade Federal do Rio Grande do Sul, Porto Alegre, RS, Brazil; ^3^School of Chemical Engineering, Ocean and Life Sciences, Dalian University of Technology, Panjin, China

**Keywords:** bioremediation, hydrocarbon degradation, sustainability, environmental pollution, microbial remediation, biodegradation

Recent advances in microbial ecology and molecular biology have illuminated the intricate processes facilitating hydrocarbon degradation and bioremediation, offering a deeper understanding of how microbial species, genetic frameworks, and metabolic routes contribute to these environmental restoration efforts. The discovery of several bacterial species that can degrade toxic pollutants holds immense potential for environmental scientists and engineers, sparking hope for a cleaner, healthier environment. Bacteria's use of aerobic and anaerobic pathways to metabolize contaminants such as hydrocarbons showcases the versatility of microbial bioremediation strategies. However, the anaerobic degradation of polycyclic aromatic hydrocarbons (PAH) by microorganisms, while documented, still requires further investigation for detailed mechanistic insights and practical application in bioremediation (Chen et al., 2023; Kumari and Das, 2023).

Thus, the intricate relationship between microbial communities and hydrocarbons, particularly in the context of environmental pollution, has been of great scientific interest. In this context, from seawater samples collected from the Caspian Sea, Griffiths et al. explored the response of indigenous microbial communities to crude oil in microcosms submitted to both oxic and hypoxic conditions. The study identified distinct microbial communities in surface and deeper waters of the Caspian Sea adapted to these varying conditions, with a particular focus on bacteria related to sulfate and nitrogen cycling in hypoxic environments. This work emphasized the versatility of microbial responses to hydrocarbon pollution and the potential for anaerobic biodegradation pathways in oil-contaminated sites.

In another example, Martinez-Varela et al. investigated the microbial communities of the cold, pristine waters of coastal Antarctica, revealing the critical role these communities in the sea-surface microlayer (SML) in degrading PAH. This study highlighted the faster PAH degradation rates in the SML compared to the subsurface layer. This process underscored the importance of particle-associated bacteria, particularly the hydrocarbonoclastic genus Pseudoalteromonas. In addition, metatranscriptome analysis revealed differentially expressed genes between communities exposed to PAH, indicating a greater abundance of genes expressed in SML, mainly attributed to Alteromonadales. Among the genes identified, PAH degradation genes and oxidative stress response genes stood out, suggesting their importance in the community's adaptation to the hostile environment. Taking these findings, the authors proposed that these microbial communities act as efficient bioreactors, pointing to the potential for leveraging similar mechanisms in bioremediation strategies.

Despite the significant successes achieved in hydrocarbon bioremediation in the laboratory, there is a pressing need to apply these microbial and genetic advancements to practical, large-scale bioremediation projects. The challenges, including time-consuming enrichment processes, byproducts with unknown toxicity, and activity inhibition at low temperatures (Chen et al., 2023), underscore the urgency and significance of this task. Focused research on understanding the metabolic pathways, genetic regulations, and practical challenges of bioremediation is crucial. The investigation must also aim to understand the toxicity of byproducts better, optimize conditions for microbial activity, and scale up successful laboratory models to field applications. In this sense, Vogt et al. introduced a novel perspective by examining the carbon and hydrogen stable isotope fractionation during the monooxygenation of short-chain alkanes by *Thauera butanivorans*. This work provided valuable insights into the biochemical mechanisms of alkane degradation. The work also demonstrated the utility of multi-element compound-specific stable isotope analysis (ME-CSIA) in tracking environmental biodegradation processes. The findings offered a promising tool for assessing the fate of hydrocarbons in natural and contaminated environments.

In addition, Vogel et al. challenged the assumption that the expression of critical functional genes could serve as reliable markers for PAH biodegradation. By studying *Cycloclasticus pugeti*i strain PS-1, the authors showed that the expression of PAH-degradation marker genes was independent of PAH presence, suggesting a more complex regulatory mechanism. This study called for a cautious interpretation of transcription data and highlighted the need for a broader understanding of the genetic repertoire involved in PAH degradation.

Together, the articles above painted a picture of a dynamic and complex microbial world with the potential to mitigate hydrocarbon pollution. They highlighted the importance of understanding microbial hydrocarbon degradation at both the genetic and ecological levels, paving the way for innovative bioremediation strategies. As we continue to explore the microbial toolbox for hydrocarbon degradation, these studies offer valuable insights and tools for environmental scientists and engineers alike, aiming to harness microbial processes to clean up contaminated sites. Significant strides have been made in identifying microbial species, genetic mechanisms, and metabolic pathways for hydrocarbon degradation. However, challenges must still be overcome to bridge the gap between laboratory research and practical application. Furthermore, future developments in synthetic biology, which involves the design and construction of new biological parts, devices, and systems, meta-omics, which is the study of the collective genomes, transcriptomes, proteomes, and metabolomes of a microbial community, microbiome engineering, which is the manipulation of microbial communities to achieve a desired outcome, and bioinformatics, which is the application of computational techniques to analyze biological data, for modifying microbes present a promising path to improving the degradation capabilities of microorganisms and enabling increased tolerance to toxicity and potency for faster degradation of pollutants (Yaashikaa et al., 2022; Chen et al., 2023). These approaches could help overcome current limitations by providing a more comprehensive understanding of microbial communities and their functional potential in bioremediation.

## Author contributions

LA-L: Writing—review & editing, Writing—original draft. MV: Writing—review & editing. XZ: Writing—review & editing.
